# Efficacy and safety of percutaneous mechanical circulatory support in patients with cardiogenic shock following acute myocardial infarction: A meta-analysis of randomized controlled trials

**DOI:** 10.1097/MD.0000000000040595

**Published:** 2024-11-15

**Authors:** Muhammad Daoud Tariq, Hritvik Jain, Abdul Moiz Khan, Syeda Shahnoor, Priya Goyal, Eeshal Zulfiqar, Areeba Ahsan, Vikash Jaiswal, Mohamed Daoud, Amir Humza Sohail

**Affiliations:** a Department of Internal Medicine, Foundation University Medical College, Islamabad, Pakistan; b Department of Internal Medicine, All India Institute of Medical Sciences-Jodhpur, Jodhpur, India; c Department of Internal Medicine, Ayub Medical College, Abbottabad, Pakistan; d Department of Internal Medicine, Dow University of Health Sciences, Karachi, Pakistan; e Department of Internal Medicine, Dayanand Medical College and Hospital, Punajb, India; f Department of Internal Medicine, Larkin Community Hospital, Miami, FL; g Department of Internal Medicine, Bogomolets National Medical University, Kyiv, Ukraine; h Department of Surgery, University of New Mexico Health Sciences, Albuquerque, NM.

**Keywords:** acute myocardial infarction, cardiogenic shock, IABP, impella, intra-aortic balloon pump, meta-analysis

## Abstract

**Background:**

Cardiogenic shock (CS) is a severe complication of acute myocardial infarction (AMI) with high mortality rates. While mechanical circulatory support devices like intra-aortic balloon pump (IABP) and Impella are used to manage CS, their comparative effectiveness remains unclear. This meta-analysis aims to evaluate the safety and efficacy of Impella in the treatment of AMI-associated CS.

**Methods:**

A comprehensive literature search was performed across PubMed, EMBASE, Google Scholar, SCOPUS, and Web of Science. The primary efficacy endpoint was 6-month all-cause mortality. Secondary efficacy endpoints included 30-day mortality, major bleeding, limb ischemia, sepsis, and left ventricular ejection fraction. Pooled odds ratios (OR) and standardized mean difference (SMD) with 95% confidence intervals (CIs) were calculated using the random-effects model via Revman version 5.4. Statistical significance was determined at *P* < .05.

**Results:**

Four RCTs with a total of 442 patients were included in this meta-analysis. The pooled analysis showed that the odds of 6-month all-cause mortality were significantly lower with Impella compared to standard of care (OR: 0.64, 95% CI: 0.43–0.95; *P* value: .03). However, 30-day mortality reported no statistically significant difference between the 2 groups (OR: 1.03; 95% CI: 0.43–2.48; *P* = .95). Our analysis found that the use of impella is associated with a statistically significant increase in the odds of major bleeding (OR: 3.61; 95% CI: 1.14–11.40; *P* = .03), limb ischemia (OR: 4.91; 95% CI: 1.37–17.59; *P* = .01), and sepsis (OR: 2.75; 95% CI: 1.25–6.08; *P* = .01). No statistical significance was found in left ventricular ejection fraction at follow-up between the 2 groups (SMD: −0.35; 95% CI: −0.78 to 0.07; *P* = .11).

**Conclusion:**

Impella significantly reduces 6-month all-cause mortality in patients with CS following AMI compared to standard of care. However, this survival benefit is offset by a substantial increase in major bleeding, limb ischemia, and sepsis risks associated with Impella. Future large scale trials are needed to validate these findings and refine clinical guidelines for the optimal use of Impella in treating CS.

## 1. Introduction

Cardiogenic shock (CS) is a severe condition of end-organ hypoperfusion due to impaired heart function, as outlined by the American Heart Association.^[1]^ This condition is frequently linked to acute myocardial infarction (AMI), with about 5% to 15% of AMI patients developing CS, and women being more susceptible.^[2–4]^ Despite improvements in treatment, the death rate for AMI complicated by CS remains significant. CS is a major cause of in-hospital deaths among AMI patients, with mortality rates ranging from 40% to 60%.^[4–6]^

Short-term mechanical circulatory support (MCS) devices have been explored as one of the standard options to aid impaired circulation. Whereas intra-aortic balloon pump (IABP) has been regarded as the standard of care (SOC) device in this category.^[7]^ Currently, IABP has a Class IIb recommendation in American guidelines and a Class III recommendation in European guidelines.^[8,9]^ A meta-analysis of smaller studies^[10]^ and a large randomized controlled trial (RCT) did not demonstrate a significant benefit of IABP as a SOC in the context of CS following AMI.^[7,11]^

Subsequently, Impella emerged as a viable alternative for managing pump failure in CS. Receiving its first FDA approval in 2008, the Impella is a catheter-based, impeller-driven, axial-flow pump engineered to deliver up to 2.5 liters per minute of blood flow from the left ventricle to the ascending aorta.^[12]^ Recent studies have indicated that Impella provides enhanced hemodynamic support in acute settings compared to IABP.^[13,14]^ However, 2 small randomized trials in AMI patients lacked statistical power to detect clinical outcome differences.^[15,16]^ However, in the recent IMPRESS study, Impella CP did not reduce 30-day mortality but was associated with an increase in severe bleeding incidents.^[17]^ Impella 5.0 is a recently introduced subtype of MCS that has the capacity to provide a 5 L/min flow rate which enables complete left ventricular support and provides more stable hemodynamic effects on myocardial oxygen consumption. Impella 5.0 achieves an excellent safety profile as it provides better circulatory support, myocardial unloading, and axillary placement that enables early patient mobilization and rehabilitation.^[18]^

Our meta-analysis aims to assess the comparative effectiveness of Impella to SOC in the management of CS following AMI. By synthesizing data from available clinical trials, we seek to clarify their relative benefits and risks, providing updated evidence to inform clinical practice and guide future research in treating CS associated with AMI.

## 2. Methods

This meta-analysis was performed in accordance with the procedures suggested by the Cochrane Collaboration^[19]^ and adhered to the Preferred Reporting Items for Systematic Review and Meta-Analysis Statement (PRISMA) 2020 guidelines for systematic reviews and meta-analyses.^[20]^ This study was registered with the PROSPERO International Prospective Register of Systematic Reviews (CRD42024568202). The PRISMA 2020 checklist is also provided in the Supplementary File.

### 2.1. Data sources and search strategy

We carried out a comprehensive electronic search across PubMed, EMBASE, Google Scholar, SCOPUS, and Web of Science from their inception until June 2024. Our objective was to identify RCTs that evaluated the outcomes of Impella compared to the SOC). No restrictions for language or time were imposed during our search. The search strategy employed a combination of the following Medical Subject Headings (MeSH) terms and free-text keywords including “percutaneous mechanical circulatory support” or “impella” or “intra-aortic balloon pump” or “cardiogenic shock” or “acute myocardial infarction.” Boolean operators such as “AND” and “OR” were used to create a search strategy in combination with keywords. Furthermore, we manually reviewed the reference lists of the selected articles to ensure comprehensive coverage and avoid missing any pertinent studies. The detailed search strategy is attached in the Supplementary File as Table S1, Supplemental Digital Content, http://links.lww.com/MD/N974.

### 2.2. Eligibility criteria

Inclusion criteria: We established the inclusion criteria based on the PICOs format typically used in systematic reviews and meta-analyses. The population (P) comprised patients with CS as a complication of AMI. The intervention (I) involved patients receiving Impella (or MCS), while the control (C) was SOC. The outcomes (O) of interest included 6-month all-cause mortality, 30-day mortality, sepsis, limb ischemia, left ventricular ejection fraction (LVEF) at follow-up, and major bleeding events.

Exclusion Criteria: The exclusion criteria were studies that did not report our preferred outcomes and subjects without CS. Additionally, articles not in English, non-peer-reviewed publications, editorials, commentaries, case reports, case series, review articles, and meta-analyses were excluded.

### 2.3. Study selection

The EndNote Reference Manager (Version X7.5; Clarivate Analytics, Philadelphia, PA, 2016) was used to export all articles obtained from the systematic search. Two authors (M.D.T and A.M.K) independently screened the search results to identify studies that met the inclusion criteria. Full-text articles of potentially relevant studies were retrieved and assessed for final inclusion. References from previous systematic reviews and meta-analyses were also manually reviewed to ensure no significant publications were missed. Duplicate articles were removed. The 2 authors then evaluated the titles and abstracts of the remaining publications, with complete texts examined for relevancy. Any disagreements between the authors were resolved through discussion and consensus with a third author (S.S).

### 2.4. Data extraction and quality assessment

Data extraction was performed by 2 authors (M.D.T and A.M.K) independently using a pre-piloted Microsoft Excel spreadsheet. Any discrepancies at any stage were resolved by a third reviewer (S.S). Data extracted from eligible studies included the first author’s name, year of publication, study design, sample size, baseline characteristics of the study population (including age, gender, type of AAV), and reported outcomes. Quality evaluation was conducted using the modified Cochrane Collaboration risk-of-bias 2.0 tool specifically designed for RCTs.^[21]^

### 2.5. Outcomes of interest

Our study aimed to assess the primary outcome of 6-month all-cause mortality. In addition, we examined secondary outcomes, including 30-day mortality, major bleeding, limb ischemia, sepsis, and LVEF.

### 2.6. Data synthesis

The data was synthesized using Cochrane Review Manager software (RevMan version 5.4.1). A random-effects model was applied, utilizing odds ratio (OR) and standardized mean difference (SMD) as the effect measures, with 95% confidence intervals (CIs) and statistical significance determined at *P* < .05 for pooling the results of individual studies. The heterogeneity among the studies was assessed using Higgin’s *I*^2^ test, with *I*^2^ values interpreted as follows: 0% to 25% indicating low heterogeneity, 25% to 75% indicating moderate heterogeneity, and >75% indicating high heterogeneity. Influential studies affecting heterogeneity were identified using leave-one-out sensitivity analysis. Publication bias was examined through visual inspection of funnel plots.

## 3. Results

### 3.1. Study selection

**Figure 1.**
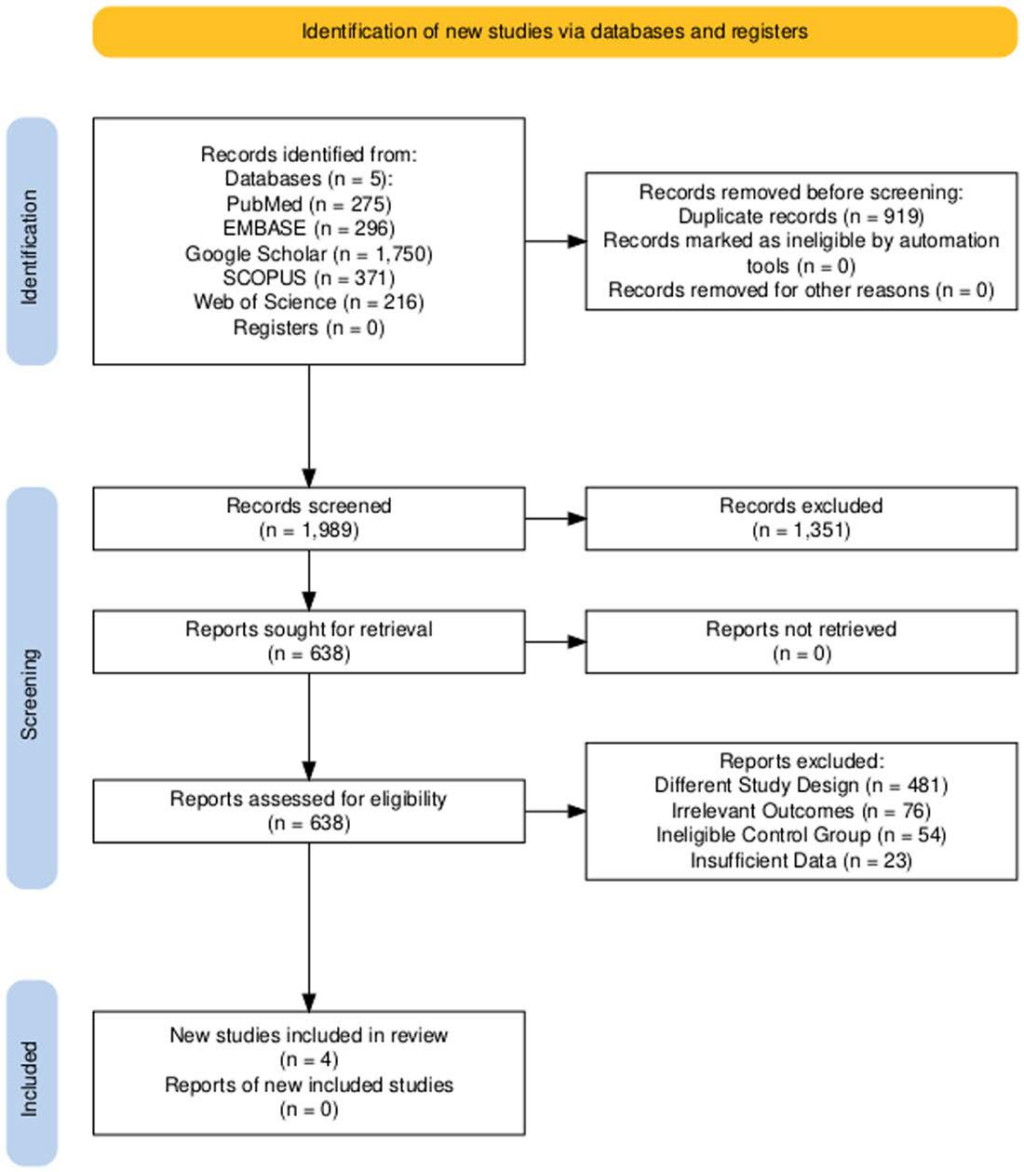
The 2020 preferred reporting items for systematic reviews and meta-analyses flowchart.

A total of 2908 records were identified from various databases. After the initial screening, 919 articles were excluded due to data duplication or because their titles and abstracts did not meet our inclusion criteria. This left 638 studies, which were assessed in full text after excluding those that did not meet the inclusion criteria. Further screening led to the exclusion of 481 due to different study designs, 76 articles due to irrelevant outcomes, 54 due to ineligible control groups, and 23 due to insufficient data. Ultimately, 4 studies met our criteria and were included in the meta-analysis. The detailed steps of our literature search are depicted in the PRISMA flow chart (Fig. 1).

### 3.2. Study and patient characteristics

Following an extensive screening process, we included 4 RCTs in our meta-analysis.^[15,17,22,23]^ These studies comprised a total of 442 patients, with 223 in the intervention group and 219 in the control group. Detailed baseline characteristics of these studies are presented in Table 1. The mean age of participants in the intervention and control groups were 62.40 ± 11.59 and 62.12 ± 12.76 years, respectively. A summary of the patients’ baseline characteristics is shown in Table 2.

**Table 1:** Study baseline characteristics

Study ID	Country of origin	Study design	Participants (n)		Age, years (Mean ± SD)		Males (n)		Inclusion criteria	Exclusion criteria
			Impella	SOC	Impella	SOC	Impella	SOC		
Bochaton 2020^[22]^	France	Randomized Controlled Trial (RCT)	7	6	60.3 ± 12.3	53.5 ± 8.1	6	6	Patients admitted with CS-AMI, who had been treated with primary angioplasty within 24 h of the index AMI, and required inotropic drugs and an IABP, were eligible for inclusion	Patients that had a contraindication to Impella implantation (aortic valvulopathy or mechanical valve, hypertrophic cardiomyopathy, left ventricular thrombus), were in refractory cardiogenic shock (INTERMACS 1 or 2; high dose of norepinephrine or any dose of epinephrine), had right ventricular failure, had been resuscitated for cardiac arrest for > 30 min or had a septic condition
Møller 2024^[23]^	Denmark, Germany, and the United Kingdom	Randomized Controlled Trial (RCT)	179	176	67 ± 13.45	68.67 ± 11.21	142	139	Patients 18 years of age or older with STEMI and cardiogenic shock. Cardiogenic shock was defined as hypotension (systolic blood pressure below 100 mm Hg or an ongoing need for vasopressor support), end-organ hypoperfusion with an arterial lactate level of 2.5 mmol per liter or greater, and a left ventricular ejection fraction of less than 45%	Patients who had been resuscitated from out-of-hospital cardiac arrest and remained comatose on arrival to the cardiac catheterization laboratory and patients with overt right ventricular failure were excluded
Ouweneel 2017^[17]^	Netherlands	Randomized Controlled Trial (RCT)	24	24	58 ± 9	59 ± 11	18	20	Patients presented with an AMI with ST segment elevation complicated by severe CS in the setting of immediate percutaneous coronary intervention (PCI). Severe CS was defined as a systolic blood pressure <90 mm Hg for longer than 30 min or the need for inotropes or vasopressors to maintain a systolic blood pressure > 90 mm Hg	Severe aorto-iliac arterial disease impeding placement of either IABP or pMCS, known severe cardiac aortic valvular disease, serious known concomitant disease with a life expectancy of <1 year, known participation in this study or any other trial within the previous 30 d, or coronary artery bypass grafting within the preceding week
Seyfarth 2008^[15]^	Germany	Randomized Controlled Trial (RCT)	13	13	64.33 ± 11.63	67.33 ± 20.76	8	11	Patients with: acute myocardial infarction (AMI) < 48 h, confirmed by ischemic symptoms for at least 30 min with elevated cardiac markers or ST-segment elevation or left bundle branch block. An AMI was suspected when patients were resuscitated, and cardiac markers and/or electrocardiographic changes met criteria for AMI/acute coronary syndrome; Cardiogenic shock was defined using both clinical and hemodynamic criteria as previously described in the SHOCK trial	Age < 18 years; prolonged resuscitation (>30 min); hypertrophic obstructive cardiomyopathy; definite thrombus in left ventricle; treatment with IABP; severe valvular disease or mechanical heart valve; cardiogenic shock caused by mechanical complications of AMI such as ventricular septal defect, acute mitral regurgitation greater than second degree, or rupture of the ventricle; predominant right ventricular failure or the need for a right ventricular assist device; sepsis; known cerebral disease; bleeding with a need for surgical intervention; pulmonary embolism; allergy to heparin or any known coagulopathy; aortic regurgitation greater than second degree; pregnancy; or inclusion in another study or trial

AMI = acute myocardial infarction, CS = cardiogenic shock, IABP = intra-aortic balloon pump, PCI = percutaneous coronary intervention, pMCS = percutaneous mechanical circulatory support, SOC = standard of care, STEMI = ST elevation myocardial infarction.

**Table 2:** Patients baseline characteristics

Study ID	Heart rate, beats/min (mean ± SD)		Left ventricular ejection fraction, % (mean ± SD)		Mean arterial pressure, mm Hg (mean ± SD)		Systolic blood pressure, mm Hg (mean ± SD)		Diastolic blood pressure, mm Hg (mean ± SD)		Hypertension, n/N		Diabetes mellitus, n/N		Previous MI, n/N		Peripheral arterial disease, n/N		Prior PCI or CABG, n/N	
	Impella	SOC	Impella	SOC	Impella	SOC	Impella	SOC	Impella	SOC	Impella	SOC	Impella	SOC	Impella	SOC	Impella	SOC	Impella	SOC
Bochaton 2020^[22]^	98.1 ± 27.6	103.8 ± 14.4	29 ± 6	30 ± 8	67.7 ± 12.3	69.9 ± 7.8	NR	NR	NR	NR	NR	NR	2/7	0/6	1/7	0/6	NR	NR	NR	NR
Møller 2024^[23]^	93.67 ± 24.66	94.00 ± 26.16	25.33 ± 8.22	23.33 ± 11.21	63.33 ± 12.71	64.00 ± 13.45	82.33 ± 14.20	81.67 ± 14.20	NR	NR	89/179	94/176	33/179	47/176	29/179	28/176	NR	NR	NR	NR
Ouweneel 2017^[17]^	81 ± 21	83 ± 28	NR	NR	66 ± 15	66 ± 15	81 ± 17	84 ± 19	58 ± 22	57 ± 13	4/20	6/21	2/22	3/23	1/22	1/23	2/23	0/23	1/22	0/23
Seyfarth 2008^[15]^	95 ± 24	97 ± 24	28.67 ± 15.78	31.67 ± 17.44	78 ± 16	72 ± 17	106 ± 22	101 ± 23	64 ± 15	58 ± 14	7/13	9/13	5/13	3/13	NR	NR	NR	NR	12/13	13/13

CABG = coronary artery bypass grafting, MI = myocardial infarction, PCI = percutaneous coronary intervention, SOC = standard of care.

### 3.3. Endpoints

**Figure 2.**
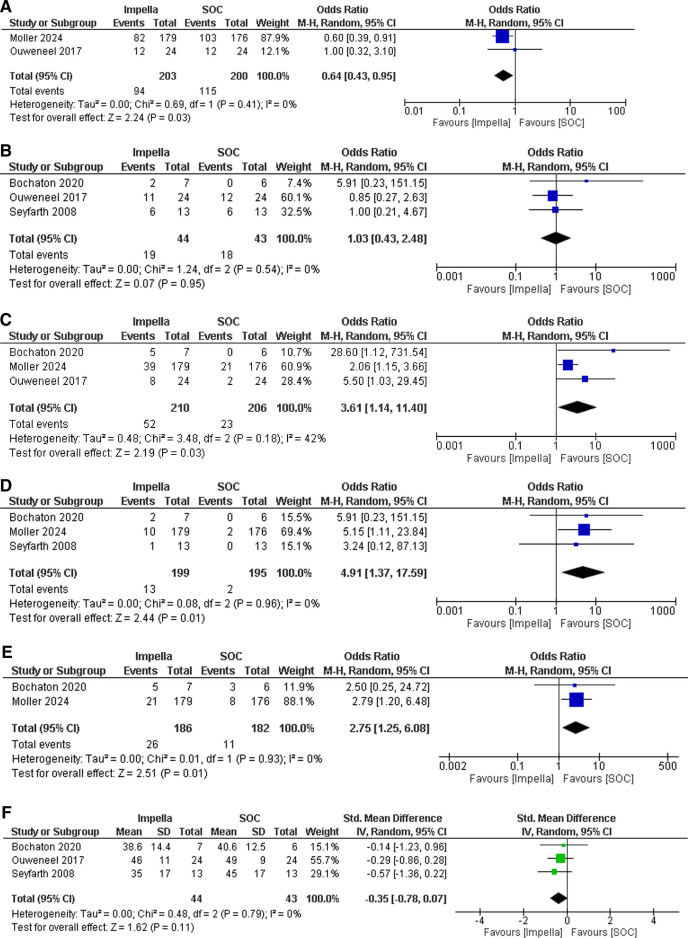
Individual and pooled analyses illustrating the efficacy and safety of impella compared to standard of care (SOC) in cardiogenic shock patients complicated by acute myocardial infarction. The odds ratio (OR) and standardized mean difference (SMD) with their 95% confidence intervals (CIs) are displayed using a logarithmic scale, with the box size scaling in accordance with the sample size. The diamond symbolizes the combined or overall effect.

Two studies reported 6-month all-cause mortality as an outcome.^[17,23]^ The pooled analysis found Impella to significantly reduce the odds of 6-month all-cause mortality in comparison to SOC (OR: 0.64; 95% CI: 0.43–0.95; *P* = .03; *I*^2^ = 0%) (Fig. 2A). No heterogeneity was found among the studies. Three studies documented the outcome of 30-day mortality.^[15,17,22]^ Our analysis reported no statistically significant difference between the 2 groups (OR: 1.03; 95% CI: 0.43–2.48; *P* = .95; *I*^2^ = 0%) (Fig. 2B). No heterogeneity was observed among the studies.

The outcome of major bleeding was part of 3 studies.^[17,22,23]^ Our analysis found the use of impella to increase the odds of bleeding by more than 3 times as compared to SOC and the results were statistically significant (OR: 3.61; 95% CI: 1.14–11.40; *P* = .03; *I*^2^ = 42%) (Fig. 2C). Moderate heterogeneity was reported among the studies. Upon performing the leave-one-out sensitivity analysis, the heterogeneity was reduced to zero after excluding Møller et al.^[23]^ Three studies reported data on limb ischemia.^[15,22,23]^ Our analysis revealed impella to increase the odds of limb ischemia by nearly 5 times as compared to SOC and the results were statistically significant (OR: 4.91; 95% CI: 1.37–17.59; *P* = .01; *I*^2^ = 0%) (Fig. 2D). No heterogeneity was found among the studies.

Sepsis as an outcome was included in 2 studies.^[22,23]^ Our analysis revealed the use of impella to increase the sepsis odds by more than 2 times as compared to SOC and the results were statistically significant (OR: 2.75; 95% CI: 1.25–6.08; *P* = .01; *I*^2^ = 0%) (Fig. 2E). No heterogeneity was noticed among the studies. Three studies reported data on LVEF at follow-up^[15,17,22]^ (Fig. 2F). Our pooled analysis showed no statistically significant difference between the 2 groups (SMD: −0.35; 95% CI: −0.78 to 0.064; *P* = .096; *I*^2^ = 0%). No heterogeneity was noticed among the studies.

### 3.4. Risk of bias and publication bias assessment

The risk of bias was “low” for most of the included studies. However, Bochaton et al^[21]^ showed “some concerns” (Figure S1, Supplemental Digital Content, http://links.lww.com/MD/N974). On visual inspection of funnel plots, symmetrical appearance demonstrated no to low risk of publication bias (Figure S2, Supplemental Digital Content, http://links.lww.com/MD/N974).

## 4. Discussion

Our study concludes that Impella use is associated with reduction in the risk of 6-month all-cause mortality. Although the use of Impella has been associated with an increase in complications such as major bleeding, sepsis, and limb ischemia, these complications do not translate into increased mortality. These results indicate that the Impella is a highly effective device, and further advancements that help mitigate these complications could potentially enhance its overall efficacy.

A National Inpatient Sample review indicated a significant rise in Impella usage for AMI complicated by CS, increasing almost 5-fold from 4.1% in 2012 to 19.9% in 2017.^[24]^ One reason for this increased usage is Impella’s easy percutaneous approach, facilitated by its catheter-based, miniaturized rotary blood pump design.^[25]^ Institutions that have adopted these approaches have reported notable increases in survival rates.^[26]^ Studies indicate survival to discharge rates ranging from 40.7% to as high as 76% to 81.3%, reflecting substantial improvements over historical control rates.^[26–29]^ Conversely, the use of IABP as a standalone treatment in SOC has declined, following studies and guideline updates that did not support its benefit in CS.

Our meta-analysis of RCTs concluded that Impella significantly reduces 6-month all-cause mortality in CS patients, thus resolving the debate on its impact on outcomes. Our findings mirror a large meta-analysis showing improved CS mortality with Impella when other factors are controlled.^[30]^ Panuccio et al^[30]^ conducted a systematic review encompassing retrospective analyses and prospective observational studies, with a total of thirty-three studies and over 5200 CS patients; and 7 studies comparing Impella to IABP. The investigators revealed a short-term mortality of 47% with the use of the Impella device for CS, which showed patient age, higher support level, and pre-PCI insertion as significant moderators in meta-regression. Additionally, when compared to IABP, Impella demonstrated no significant difference in short-term mortality (RR: 1.08; 95% CI: 0.89, 1.31), which is in line with the current RCT-only meta-analysis (OR: 1.03, 95% CI: 0.43, 2.48). Furthermore, Panuccio et al^[30]^ also reported a significant increase in major bleeding with Impella compared to IABP (RR: 1.99, 95% CI: 1.75, 2.25; *P* < .00001), which is again in line with our study (OR: 3.61, 05% CI: 1.14, 11.40; *P* = .03). Including observational studies significantly increases the risk of bias through selective reporting, where only significant variables are reported in multivariate analyses, which has been acknowledged by Panuccio et al as they noted that not all included studies reported data on all pre-specified secondary endpoints. Additionally, including observational studies increases heterogeneity amongst the study variables, as evidenced by high heterogeneity (*I*^2^ = 67%) for short-term mortality by Panuccio et al,^[30]^ whereas we report no heterogeneity in short-term (30-day) mortality. Several similarities and differences are noted between the current RCT-only meta-analysis and the meta-analysis by Panuccio et al.^[30]^ The recent DanGer Shock RCT also supports Impella’s role in reducing mortality in CS.^[23]^ The meta-regression analysis indicated that higher MCS i.e Impella CP, improved mortality in CS patients, whereas lower levels of MCS i.e Impella 2.5, did not.^[30]^ While we could not perform a subgroup analysis, the RCTs in our meta-analysis that utilized Impella CP, i.e Ouweneel^[17]^ and Møller et al^[23]^ demonstrated comparable or superior mortality rates compared to those using lower MCS. This finding aligns with previous literature. Another factor that contributes to the success of impella is the timing of insertion. In our analysis, the RCT by Bochaton had the worst mortality outcomes, negatively impacting our overall findings.^[22]^ The authors attributed this to the implantation of the device post-reperfusion, which contradicted the hypothesis that early Impella implantation before reperfusion could help reduce infarct size after successful reperfusion.^[31,32]^ While the optimal timing for MCS in CS patients undergoing percutaneous coronary intervention (PCI) is unclear, meta-analysis and data from 38 US hospitals in the USpella registry suggest that early implantation of Impella before PCI improves survival.^[33,34]^ The National Cardiogenic Shock Initiative reported a 72% survival rate using a shock protocol emphasizing Impella support before PCI and PAC for hemodynamic monitoring.^[35]^ Therefore, the protocol of MCS installation before PCI, and 1.5 hours from the onset of shock need to be followed for optimal results.^[36,37]^

Our analysis also revealed that Impella substantially elevates the risk of bleeding. This is in agreement with a registry study of 78 patients that also found severe bleeding in 26% of MCS-treated patients versus 6% of those treated with IABP.^[38]^ Other studies comparing Impella to IABP reported that major bleeding was 2 to 4 times more common with Impella.^[15,39–41]^ Bleeding during Impella insertions is often due to the combined use of heparin and dual antiplatelet therapy post-PCI, especially in patients with traumatic injuries and conditions like heparin-induced thrombocytopenia and coagulopathies.^[17,42,43]^ The mortality risk from bleeding significantly depends on the bleeding site. A meta-analysis found that both access and non-access site (internal bleeding in organs) bleeding increased mortality risk, but non-access site bleeding had a much higher risk ratio (RR 4.06 vs 1.71).^[44]^

For high-risk bleeding patients, the dry closure technique with balloon tamponade is recommended. This involves inflating a balloon near the access site and gradually deflating it until hemostasis is achieved, followed by closing the site with Perclose sutures.^[45]^ New techniques like the single-access Impella method,^[46]^ and routine ultrasound guidance for large-bore femoral access^[47]^ show the potential to reduce complications. Additionally, reducing the size of the bore aims to lower the incidence of vascular and bleeding issues.^[45]^ Despite improved access site management and anticoagulation techniques, recent research on Impella-supported interventions remains limited, hindering the assessment of bleeding complication solutions. The RCTs in our meta-analysis didn’t specify bleeding sources, underscoring the need for future studies to document and analyze these factors to enhance management strategies.

Our analysis found Impella to increase the risk of sepsis 2-fold compared to SOC. The invasiveness of the Impella procedure contributes to a persistent risk of sepsis.^[48]^ Similarly, a propensity-score matched model showed that the Impella group had nearly twice the sepsis rates of the IABP group (12.69% vs 6.44%; *P* = .01).^[49]^ Sepsis is regarded as the most frequent cause of 30-day readmissions among patients who survived the initial hospitalization with CS.^[50]^ The greater risk of sepsis with Impella compared to IABP is due to the larger sheath size required by Impella.^[49]^ These devices are often inserted through a large-bore vascular access under emergency conditions.^[51]^ Impella 2.5 and Impella CP need a 13 or 14 Fr sheath, whereas IABP uses a smaller 7 or 8 Fr sheath, increasing the likelihood of vascular complications and sepsis.^[40]^ Impella insertion frequently involves femoral access, favoring the common femoral artery because of its adequate size.^[42]^ To minimize sepsis complications, the common femoral artery should be sufficiently large to accommodate a large-bore sheath and avoid unnecessary trauma, as determined by noninvasive imaging.^[52]^

Similarly, our study showed significantly higher odds of limb ischemia with Impella usage compared to SOC. The use of Impella, particularly with larger catheter sizes up to 14 Fr, has a downside. In patients with narrowed femoral arteries due to their baseline size or some anomaly, blood flow may be compromised, leading to distal leg ischemia.^[53]^ The occurrence of distal leg ischemia with Impella CP ranges from 4% to 17%.^[54,55]^ This condition is more frequently observed in females, attributed to the smaller diameter of their femoral arteries, and in older patients, likely due to underlying peripheral vascular disease.^[56]^ To prevent limb ischemia, several novel techniques have been suggested, including distal perfusion catheter placement, contralateral femoral external bypass, contralateral femoral internal bypass, ipsilateral femoral external bypass, and ipsilateral radial to femoral external bypass.^[53,57,58]^ With the rising trends in heart failure and CS in the United States,^[59]^ our results warrant further large scale RCTs that can further validate the findings of our study.

## 5. Limitations

Our meta-analysis has several limitations. First, the number of RCTs included is relatively small, which may impact the generalizability of our findings. A majority of the patients analyzed were derived from one recent RCT, potentially skewing the meta-analysis results to align closely with those of that individual trial. The studies also did not provide detailed categorizations of bleeding sources, limiting our ability to analyze specific bleeding risks comprehensively. Moreover, the inability to perform subgroup analyses due to limited data restricts our understanding of how different patient subgroups may benefit differently from Impella. Also, the baseline characteristics of the participants could not be adjusted for in this meta-analysis, hence we could not identify potential confounders. Finally, potential publication bias may have influenced our results. Future research should aim to address these limitations by including larger, well-designed RCTs with standardized reporting of outcomes and more granular data on complications.

## 6. Conclusion

Our meta-analysis reveals that Impella significantly reduces 6-months all-cause mortality in patients with CS following AMI compared to SOC. However, this survival benefit is offset by a substantial increase in major bleeding, limb ischemia, and sepsis risks associated with Impella. These findings highlight the need for careful patient selection, stringent management of potential complications, and further advancements in device technology and procedural protocols. Despite higher complication rates, Impella’s effectiveness in reducing mortality suggests it holds promise as a preferred MCS device in critical care settings. Future research should focus on large-scale, well-designed RCTs to validate these findings and refine clinical guidelines for the optimal use of Impella in treating CS.

## Acknowledgments

The authors have no acknowledgments to declare.

## Author contributions

**Conceptualization:** Muhammad Daoud Tariq, Amir Humza Sohail.

**Formal analysis:** Muhammad Daoud Tariq, Areeba Ahsan.

**Methodology:** Abdul Moiz Khan, Syeda Shahnoor.

**Project administration:** Muhammad Daoud Tariq, Hritvik Jain, Mohamed Daoud, Amir Humza Sohail.

**Resources:** Eeshal Zulfiqar.

**Writing – original draft:** Muhammad Daoud Tariq, Abdul Moiz Khan, Syeda Shahnoor, Priya Goyal, Eeshal Zulfiqar, Areeba Ahsan, Vikash Jaiswal.

**Writing – review & editing:** Hritvik Jain, Priya Goyal, Vikash Jaiswal, Mohamed Daoud, Amir Humza Sohail.

## Supplementary Material
