# Supplementary material for: Efficacy and safety of percutaneous mechanical circulatory support in patients with cardiogenic shock following acute myocardial infarction: A meta-analysis of randomized controlled trials
Source: Medicine (Baltimore). 2024 Nov 15;103(46):e40595. doi: 10.1097/MD.0000000000040595 (PMC11576003; doi:10.1097/MD.0000000000040595)
Supplement: Supplementary file 1 [file medi-103-e40595-s001.pdf]

Table S1: Search Strategy

No.	Database	Search Strategy	No. of articles
1	PubMed	(((("Shock, Cardiogenic"[Mesh] OR "cardiogenic shock" OR "CS") AND ("Myocardial Infarction"[Mesh] OR "MI" OR "heart attack") AND ("Intra-Aortic Balloon Pumping"[Mesh] OR "intra-aortic balloon pump" OR "IABP" OR "intraaortic balloon counterpulsation") AND ("Impella" OR "percutaneous ventricular assist device" OR "PVAD")))).	275
2	Embase	('cardiogenic shock'/exp OR 'cardiogenic shock' OR 'CS') AND ('myocardial infarction'/exp OR 'myocardial infarction' OR 'heart attack' OR 'MI') AND ('intra aortic balloon pump'/exp OR 'intra-aortic balloon pump' OR 'IABP' OR 'intraaortic balloon counterpulsation') AND ('impella'/exp OR 'Impella' OR 'percutaneous ventricular assist device' OR 'PVAD')	296
3	Google Scholar	("Cardiogenic Shock") AND ("Myocardial Infarction" OR "heart attack") AND ("Intra-aortic balloon pump" OR "IABP" OR "intraaortic balloon counterpulsation") AND ("Impella" OR "percutaneous ventricular assist device" OR "PVAD") AND ("randomized controlled trial" OR "RCT")	1750
4	Scopus	(TITLE-ABS-KEY("cardiogenic shock") AND TITLE-ABS-KEY("myocardial infarction" OR "heart attack") AND TITLE-ABS-KEY("intra-aortic balloon pump" OR "IABP" OR "intraaortic balloon counterpulsation") AND TITLE-ABS-KEY("Impella" OR "percutaneous ventricular assist device" OR "PVAD"))	371
5	Web of Science	TS=("cardiogenic shock") AND TS=("myocardial infarction" OR "heart attack") AND TS=("intra-aortic balloon pump" OR "IABP" OR "intraaortic balloon counterpulsation") AND TS=("Impella" OR "percutaneous ventricular assist device" OR "PVAD")	216

Figure 1: Risk of bias assessment of included studies utilizing Cochrane's risk of bias tool 2.0 for randomized controlled trials.

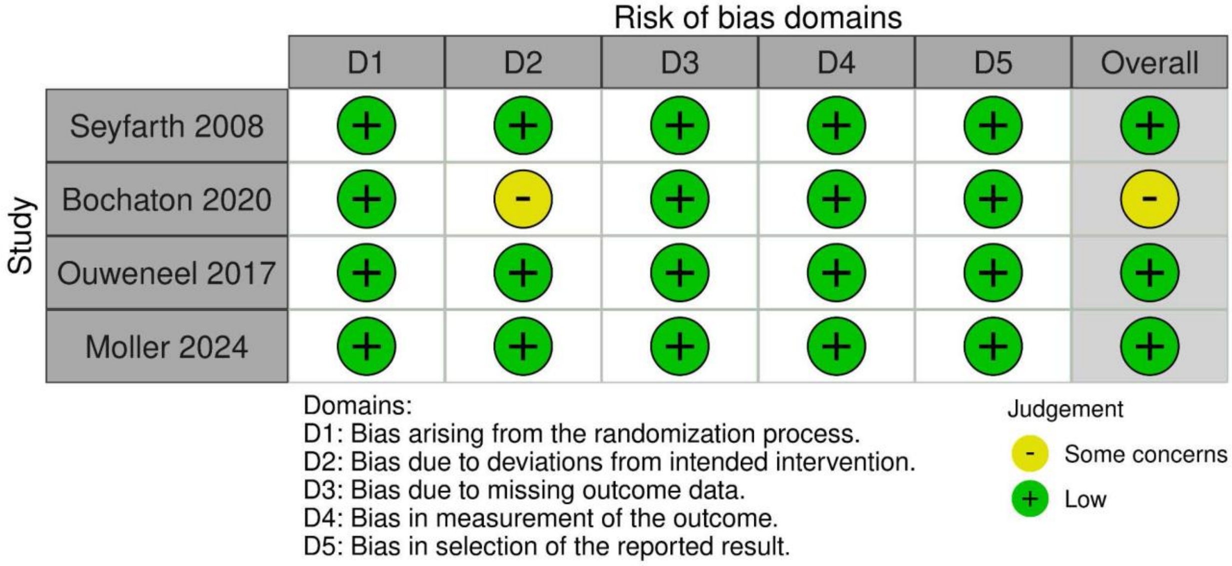

Figure 2: Funnel plots to assess for publication bias of included studies

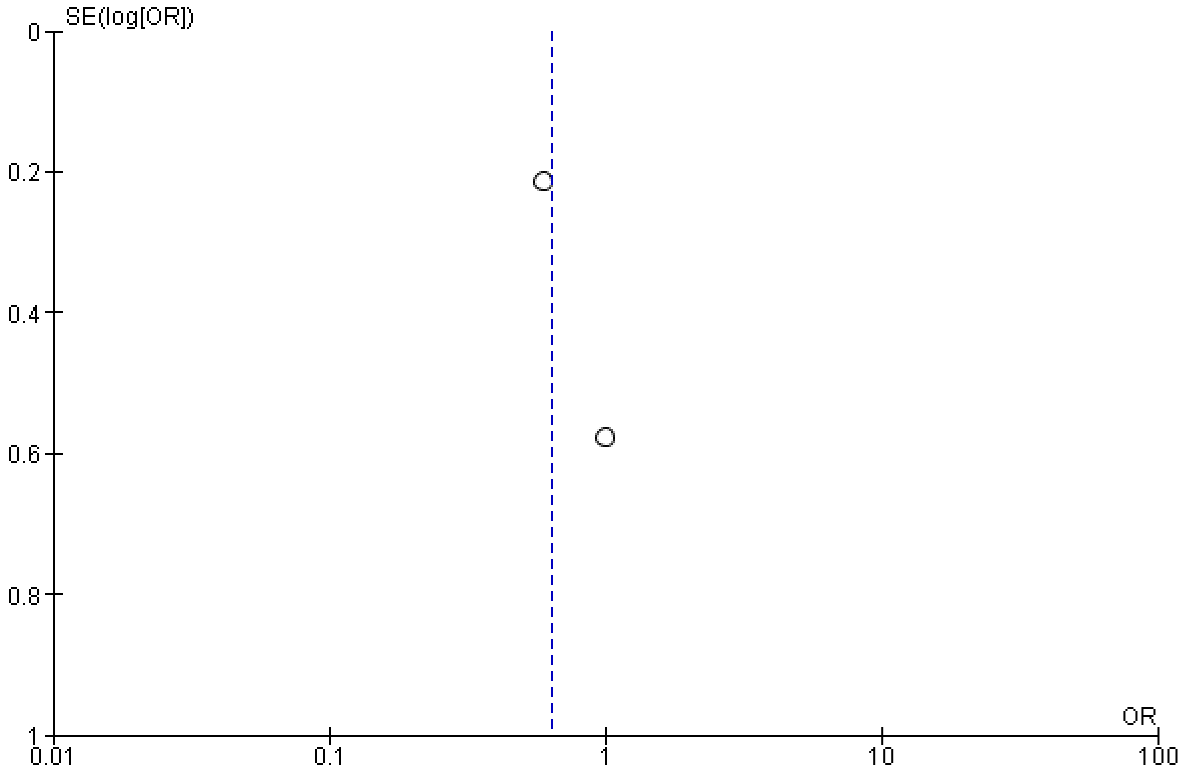

A) All-cause mortality

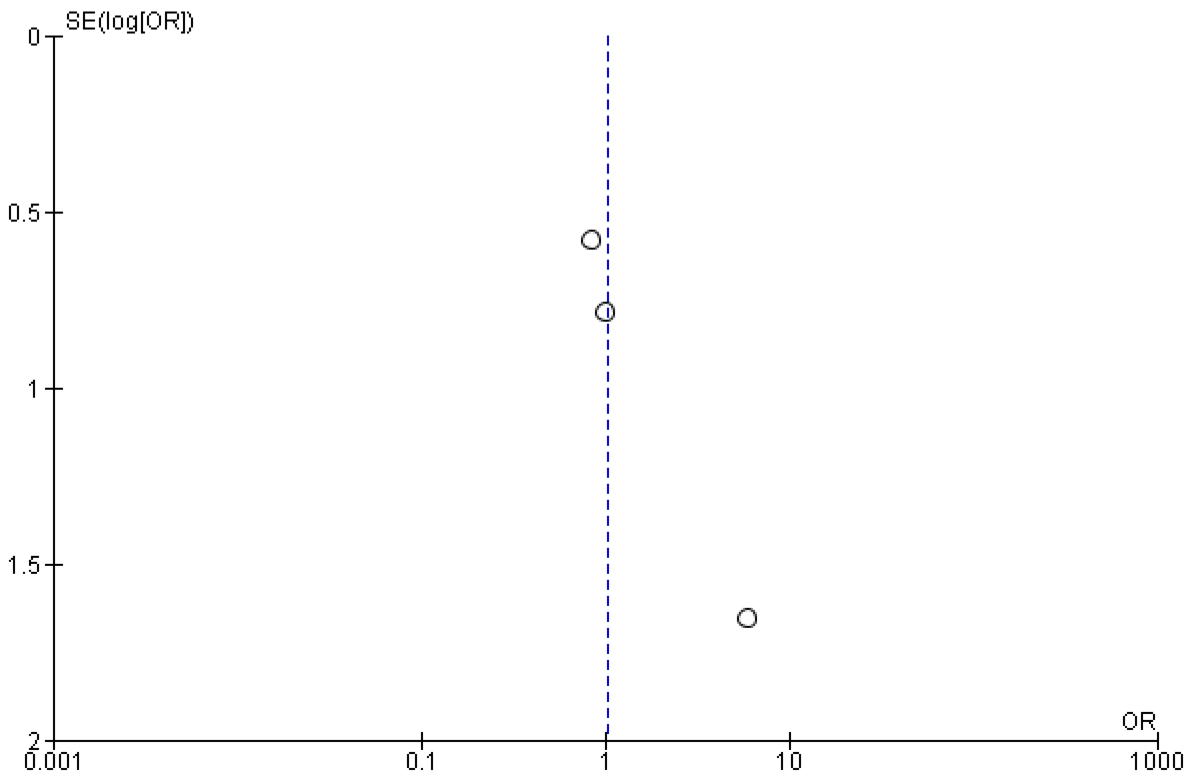

B) 30-day mortality

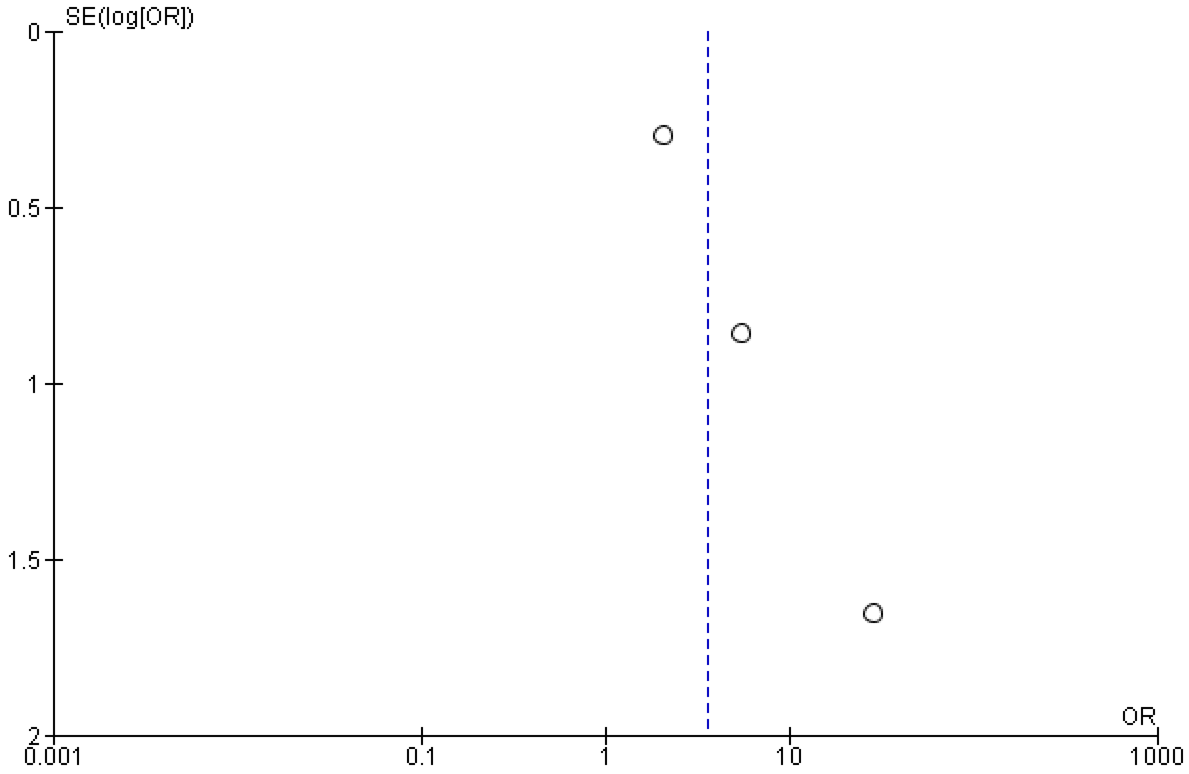

C) Major bleeding

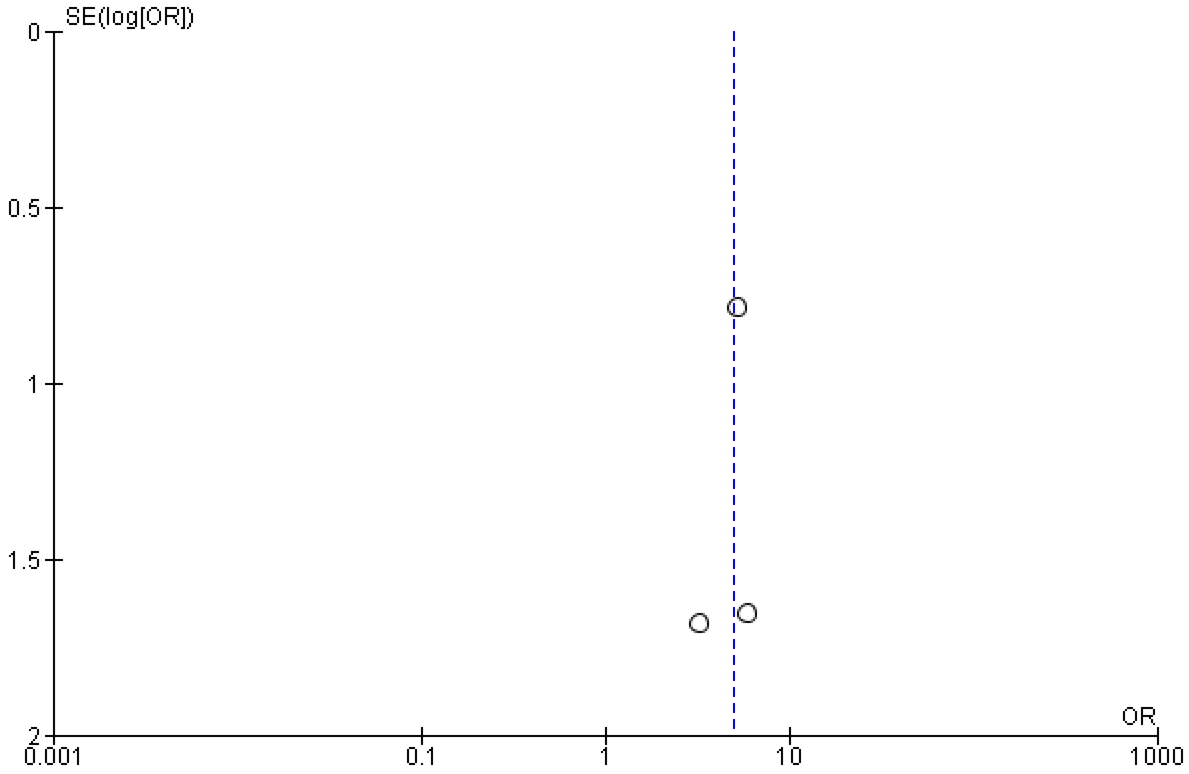

D) Limb ischemia

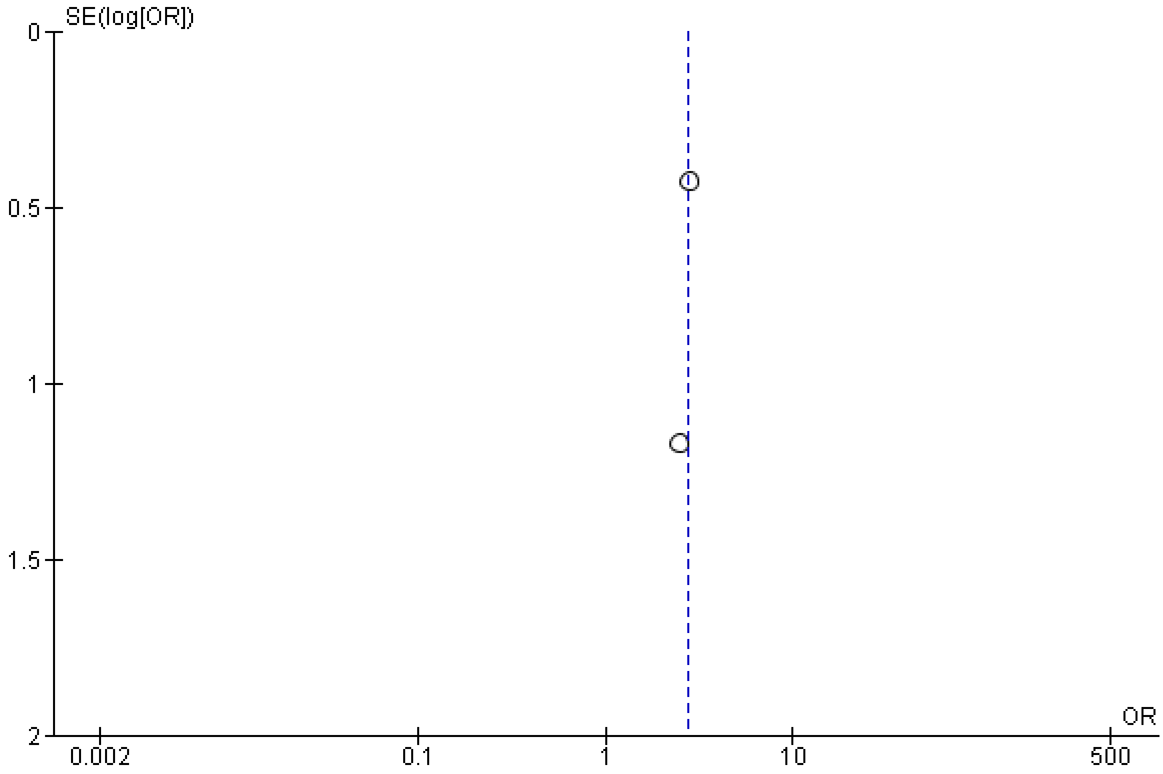

E) Sepsis

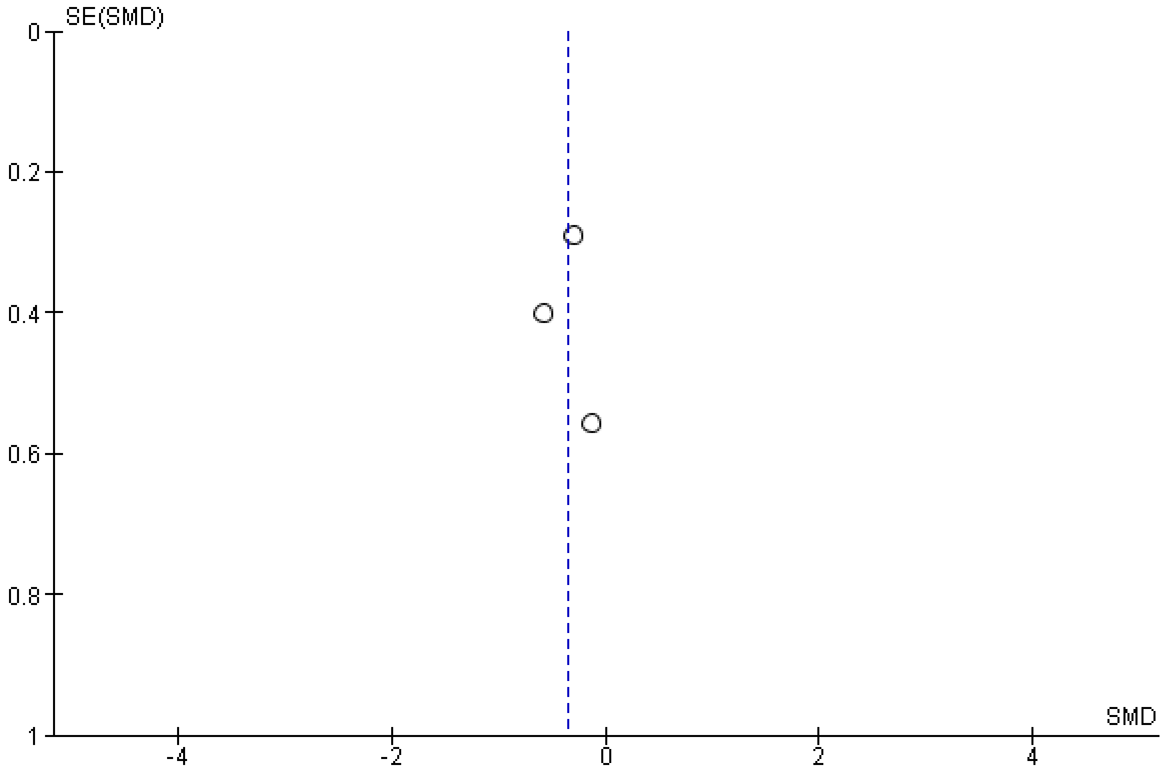

F) Left ventricular ejection fraction
